# Safeguarding the “Internet of Things” for Victim-Survivors of Domestic and Family Violence: Anticipating Exploitative Use and Encouraging Safety-by-Design

**DOI:** 10.1177/10778012231222486

**Published:** 2024-01-02

**Authors:** Andi Brown, Diarmaid Harkin, Leonie Maria Tanczer

**Affiliations:** 1 School of Social Sciences, 4919 Monash University, Melbourne, Australia; 2 Centre for Cyber Resilience and Trust, Deakin University, Waurn Ponds, Australia; 3 Department of Computer Science, 4919 University College London, London, UK

**Keywords:** technology-facilitated abuse, safety-by-design, internet of things, IoT-enabled abuse, domestic and family violence

## Abstract

Smart, Internet-connected devices—the so-called “Internet of Things” (IoT)—pose significant threats to victim-survivors of domestic and family violence (DFV). IoT systems have been used to abuse, harass, monitor, intimidate, and gaslight victim-survivors. We present findings from an abusability analysis that examined 13 IoT devices and allowed us to make several observations about common vulnerabilities to victim-survivors of DFV. We argue that IoT manufacturers must be encouraged to factor in the implications of DFV in the design of their products. Additionally, technology-facilitated abuse in DFV contexts must feature in industry and government safety-by-design approaches. Our results suggest ways IoT devices can be modified at low cost to alleviate opportunities for misuse, and we endorse IoT manufacturers to consider those risks early in the design stage.

## Introduction

An increasing amount of attention has been paid to the issue of technology-facilitated abuse (TFA) in the context of domestic and family violence (DFV). Previous studies demonstrate how a variety of digital devices are being used as a means for harassing, monitoring, intimidating, abusing, and gaslighting victim-survivors in intimate partner, parental–child, and stalking relations (Dragiewicz et al., 2018; Henry et al., 2019; Levy & Schneier, 2020; Woodlock et al., 2019). Such abuse profoundly impacts victim-survivors, often leading to depression, anxiety, suicidal ideation, and panic attacks (Mandau, 2020; Stevens et al., 2020), with digital systems playing a critical role in instances of homicide and physical abuse (Citron, 2015). The occurrence of TFA is common. For example, 99.3% of Australian frontline workers regularly assist clients, especially women and girls, who experience TFA (Woodlock et al., 2020). These figures echo dynamics observed across the globe (Messing et al., 2020; Refuge, 2020; Women's Aid, 2018).

The “Internet of Things” (IoT) potentially exacerbates this abuse angle further (Parkin et al., 2019; Tanczer et al., 2018). While conventional devices such as laptops and phones have given abusers a powerful reach, the breadth of IoT-connected systems may increase perpetrators’ influence significantly. Indeed, media reports have already illustrated how various “smart,” Internet-connected devices—such as home assistants, security cameras, lights, doorbells, and locks—are being harnessed for abusive purposes (Bowles, 2018; Riley, 2020). Faced with estimates that there will be 30 billion IoT devices by 2030 (Vailshery, 2022), we must anticipate IoT becoming an even more prominent vector for DFV-related TFA in the not-to-distant future.

To that end, this article explores the capacity to safeguard IoT products for victim-survivors of DFV through “abusability” testing and “safety-by-design” approaches. The former draws upon “usability” research to explore the ability of technologies to be abused (Strohmayer et al., 2022). The latter describes harm-preventative methods that manufacturers can use to anticipate threats or exploitative usage patterns and address them at the design stage (eSafety Commissioner, 2019). Drawing on a heuristic walkthrough framework to conduct an abusability analysis of 13 widely deployed consumer IoT devices, we uncover seven design features vulnerable to (mis)use by abusive DFV parties. To challenge the identified shortcomings, we put forward several safety-by-design suggestions that could be implemented to help safeguard consumer IoT for victim-survivors and benefit the broader consumer landscape overall.

Our article proceeds over four parts. Part one defines the concepts of TFA and IoT, showcasing how recent governmental and industrial discussions on IoT have overlooked the threat of DFV-related risks. Part two outlines the methods and enquires used to conduct an abusability analysis of our IoT “dip sample.” Part three highlights the results of our investigation and exhibits several IoT design factors that may influence the risk of IoT-facilitated DFV, as well as recommendations for their possible improvement. Finally, part four reviews the possibilities and limitations of addressing DFV-related TFA through abusability testing and safety-by-design approaches and gives pointers for industry and policy amendments.

### TFA and IoT

TFA or “tech abuse” describes how digital technologies are exploited to harass or control individuals. In the context of DFV, this digitally-enabled mode of abuse can take many forms and is frequently part of a larger abuse pattern. TFA may, thus, include threatening messages or calls, image-based abuse such as “revenge porn” (Citron & Franks, 2014; Flynn et al., 2021; McGlynn et al., 2017), as well as being tracked through phones or dedicated software such as stalkerware (Chatterjee et al., 2018; Harkin et al., 2020; Havard & Lefevre, 2020). Tech abuse is, therefore, a very ambiguous and broad concept. Activities that fall under its category range from low-tech offences to more technically sophisticated crimes, with the gaslighting element of TFA being among its most potent harm dynamics.

IoT is similarly a somewhat nebulous umbrella term. It represents novel digital systems, such as smart speakers, as well as existing objects—often appliances that were previously “offline”—which are now network-compatible. These interconnected “things” are the “direct and indirect extension of the Internet into a range of physical objects, devices, and products” (Tanczer et al., 2019, p. 37). Certain definitions of IoT emphasize that any technology containing “sensors” and an ability to communicate with other digital systems would fall under the IoT category (see, e.g., Whitmore et al., 2015). However, this definition is broad and would include ordinary devices such as smartphones or personal computers. To not dilute the term's analytical depth, our article focuses on the more novel, Internet-connected consumer items (i.e., fridges, toys, and thermostats) commonly associated with the prefix “smart.”

Thus far, several scholars and a selected number of DFV support organizations have begun to explicitly address the intersection points between DFV, TFA, and IoT (Janes et al., 2020; Leitão, 2019; Mayhew & Jahankhani, 2020; Parkin et al., 2019; Rodríguez-Rodríguez et al., 2020; Stephenson et al., 2023). Consumer IoT devices offer a concerning set of coercive and controlling behavior opportunities. They present avenues to remotely manage the access, usage, and experience of physical spaces and one's ability to escape violent environments (Bowles, 2018; Slupska & Tanczer, 2021). Furthermore, the DFV advocacy sector faces growing technical skills and knowledge shortages (Tanczer et al., 2021). Support workers struggle to understand and respond to different forms of TFA and feel they lack the adequate training and resources to effectively adjust to the shifting demands caused by the incessant tech developments (Freed et al., 2017; Lopez-Neira et al., 2019; Straw & Tanczer, 2023).

DFV advocacy organizations such as Australia's Women's Services Network (WESNET) emphasize the need to “empower” women to “take back control of their tech” (WESNET, 2020). The goal is to encourage digital participation instead of promoting avoidance of digital systems (which has become impossible in the contemporary world). In that respect, IoT design should support women and victim-survivors of DFV to enjoy the benefits and advantages of IoT safely and do so without carrying a disproportionate level of risk.

Consequently, technology companies have an essential role to play in supporting victim-survivors through prevention. Dealing with threats upstream before they result in abuse would be hugely beneficial; more efficient; and, in the context of support shortcomings, are of key strategic importance (see, e.g., Douglas, 2012; Goodman-Delahunty & Crehan, 2016; Harris & Woodlock, 2019). The role of social media and telecommunication companies has already been identified as critical to managing TFA (Dragiewicz et al., 2018; Suzor et al., 2019). This article wants to add IoT manufacturers to that network of responsibility.

### Safety-by-Design Approaches

In light of the intersections between IoT, TFA and DFV, the abusability of IoT devices is of major relevance, and the prevention of IoT (mis)use in the context of DFV should be considered in alignment with the more common aims of ensuring “security,” “privacy,” and “safety.” To define the significance of these protections more clearly: *security* ensures the device's integrity; *privacy* protects the information it contains; and *safety* aims to prevent harm. Unfortunately, these three concepts are often conflated in design approaches, resulting in the flawed assumption that improving security and/or privacy will inevitably improve safety (Brown, 2023; Strohmayer et al., 2022).

Commentators often talk about “security-by-design” (Logicworks, 2017) or “privacy-by-design” (OConnor et al., 2019) and treat safety as a byproduct of those considerations. Two significant examples of these practices include adopting two-factor authentication (TFA) as a “secure default” and increased “visibility and transparency” for privacy through the implementation of the European Union General Data Protection Regulation. There is also a strong emphasis on technical elements within security and privacy approaches. For example, the IoT Alliance Australia (IOTAA) published a *Security Guideline* that focuses on elements such as securing “routing settings,” the “TLS (transport layer security) configurations” on devices, and the security of “hardware” components (IOTAA, 2017). While these issues are essential to providing trustworthy IoT devices, purely technical solutions can overlook the unique safety concerns that arise from intimate partner violence.

Emphasizing additional safety omission, it must be noted that the (mis)use of IoT in the context of DFV has not been highlighted by the national code of practice for IoT designers (Department for Digital, Culture, Media and Sport, 2018) or the most recent Product Security and Telecommunications Infrastructure Act 2022 in the United Kingdom, the Cybersecurity Act by the European Union (European Commission, 2017), nor the voluntary code of practice for Australia (Department of Home Affairs, 2020). Therefore, while government and industrial discussions around cyber security and IoT raise several critical and timely points, the significant impact on DFV remains underappreciated (Datta Burton et al., 2021).

Instead, most IoT-centered developments focus on threats from third parties, including criminals, commercial rivals, governments, and other malicious actors (see, e.g., OConnor et al., 2019). While these are legitimate fears, the historical overemphasis on extrinsic threat actors ignores risks posed by intimate partners. The latter can rely upon physical access to devices and victim-survivors, plus intricate personal knowledge of affected parties and their systems. The threat profile of an intimate abuser is, therefore, significantly different to the unknown attacker who may exploit technical vulnerabilities for sport (Boggan 2020). Additionally, they are commonly highly motivated and determined, and frequently (mis)use legitimate functionalities and intentional design features (e.g., remote access/control; Freed et al., 2017; Stephenson et al., 2023). Therefore, the design of consumer IoT and its “user interface” (i.e., the elements with which an end-user interacts and controls a system) matters.

Existing safety-by-design approaches aimed at online platforms, such as those by the Australian eSafety Commissioner (eSafety Commissioner, 2019) or the U.K. Government (Department for Science, Innovation and Technology and Department for Digital, Culture, Media & Sport, 2021), focus on reducing harm for individuals. They put forward principles (e.g., user empowerment, autonomy, transparency, and accountability) upon which companies should orient themselves. These principles include suggestions such as setting systems to the most secure privacy and safety levels by default, providing built-in support and notification functions, and offering simple reporting routes. However, while most of these principles apply to IoT, they are not explicitly aimed at the smart consumer market, nor have the affordances provided by IoT products been tested as a means through which abuse is leveraged. Consequently, it is imperative that the IoT community starts to consider the abusability of its products and is actively encouraged to develop and apply safety-by-design frameworks.

### The Present Study

Considering the above concerns, this article offers an abusability analysis of 13 widely deployed consumer IoT devices. In addition, it reflects upon potential safety-by-design mechanisms that governments and industry actors could address. The overall goal was to probe the functioning of various smart devices and try to gauge their threat profile from the perspective of intimate partner abuse. Accordingly, smart products were tested from the point of view of whether the technology could be used abusively in the context of DFV. In doing so, the research aimed to identify risky choices that tech developers utilize within their devices, with our study offering suggestions on how best to mitigate these risks moving forward.

## Method

### Devices and Sampling

Between January 2020 and April 2020, the research team explored a dip sample of 13 commercially available IoT devices. The sample size was constrained by budget, and choices skewed toward products that appeared to be most popular within the offerings of major Australian technology stores such as JB Hi-Fi and Harvey Norman. We looked at the functioning of two popular smart home assistants, three different brands of home security cameras, two brands of smart doorbells, a smart air conditioner, a smartwatch, two different brands of fitness trackers, a smart lightbulb, and an item-tracking device (see Table 1). The sample is spread across the four main categories of smart home products identified by Altujjar and Mokhtar (2018).

**Table 1.** Devices Examined as Part of the Dip Sample of Commercially Available IoT Devices.

Name of devices examined:	Description/category:
Google Nest Hub Max	Smart home assistant/convenience
Amazon Echo Show	Smart home assistant/convenience
Tile Mate Pro Bluetooth Tracker	Item-finding device/convenience
Google Nest Cam Outdoor Security Camera	Camera/security system
EUFY Wire-Free HD Security Cam with Home Base	Camera/security system
TP-Link Kasa Pan Tilt 1080p Smart Camera	Camera/security system
Ring Video Doorbell 2	Doorbell/security system
Swann Wire-Free 720p HD Smart Video Doorbell Kit	Doorbell/security system
SPACETALK Kids Smartwatch with Phone and GPS	Kids watch/monitoring well-being
Fitbit Inspire HR	Fitness tracker/monitoring well-being
Samsung Galaxy GearFit2 Pro	Fitness tracker/monitoring well-being
Sensibo Air Conditioner and Heat Pump Wifi Controller	AC activator/energy management
Phillips Hue (Lighting)	Lightbulb/energy management

### Analysis

We analyzed the devices through an adjusted version of a “heuristic walkthrough framework,” considering victim-survivor and perpetrator perspectives, akin to Parkin et al*.* (2019). Heuristic evaluations are usability inspection methods (Ling & Salvendy, 2005). They form an adoptable instrument commonly used in human–computer interaction research. They combine techniques to detect usability problems—often in computing systems—based on prior identified enquiries (e.g., visibility, efficiency, aesthetic, user control, etc.). For example, the method has been used to test the accessibility barriers of websites (Braga et al., 2014), determine design guidelines for mobile health apps (Morey et al., 2019), and identify video game usability issues (Pinelle et al., 2008).

In our study, the method enables interrogating the *abusability* of IoT devices (see Strohmayer et al., 2022). While traditional heuristic investigations focus on how easy it is for someone to *use* technology (Sears, 1997), our study asked how easy it is for someone to *(mis)use* IoT devices for intimate partner abuse. Thus, for our abusability analysis, we explicitly looked for smart products’ capacity to be exploited, which Stephenson et al. (2023) most recently described as their “abuse vector.” We consequently probed legitimate functionalities embedded in an IoT system. Hence, devices were not subjected to penetration testing, traffic analysis, or any professional-level cyber-security vulnerability assessment. Instead, the research involved engaging directly with an IoT device's user interface and features (i.e., following manufacturers’ instructions to set up and use the devices) while systematically critiquing the process and outcomes in terms of their potential abuse in the context of DFV.

As per the requirement of the heuristic walkthrough method (Sears, 1997), a set of enquiries was developed to guide the interrogation of each unique IoT device (see Box 1 for an example). These questions were developed based on the usability heuristics employed in the literature (Parkin et al., 2019), guided by an advisory board associated with the research project (which involved domestic abuse practitioners), and prompted by the authors’ experiences. The potential for abuse—as directed by the enquiry questions—was examined using a hands-on approach with the IoT devices. Throughout the abusability analysis, the same two authors tested the devices while keeping detailed notes of the setup and operating processes. We also gathered supplementary material, including screenshots/photos of the device functioning, information from the manufacturer, and online resources (e.g., help forums and articles related to device usage). The intention of this approach was to explore the minutiae of device processes since seemingly innocuous features may increase abusability.

Box 1. Example of enquiry question guide.

**Table:** 

Device details:	
Admin account credentials:	Method of account access:
Secondary account credentials:	Method of account access:
Notes and screenshots of process/es:	
Functions: What function/s does the device perform? How easy is it to set-up the device?	
Consent: What is the process for consent? Can consent be revoked? Is authority/control clear?	
Data: What data is collected? With whom (or what) is the data shared? What notifications are provided to users about the sharing of this data? Are there logs of who accessed the data?	
Surveillance: What surveillance capabilities does the device have? How conspicuous is the device? Can the device be deployed in stealth? Is surveillance potential restricted in any way?	
Recourse: What steps could remedy abuse issues? Is there any option of recourse with the manufacturers of the technology in the event an individual was being abused with their product?	

**Figure 1.**
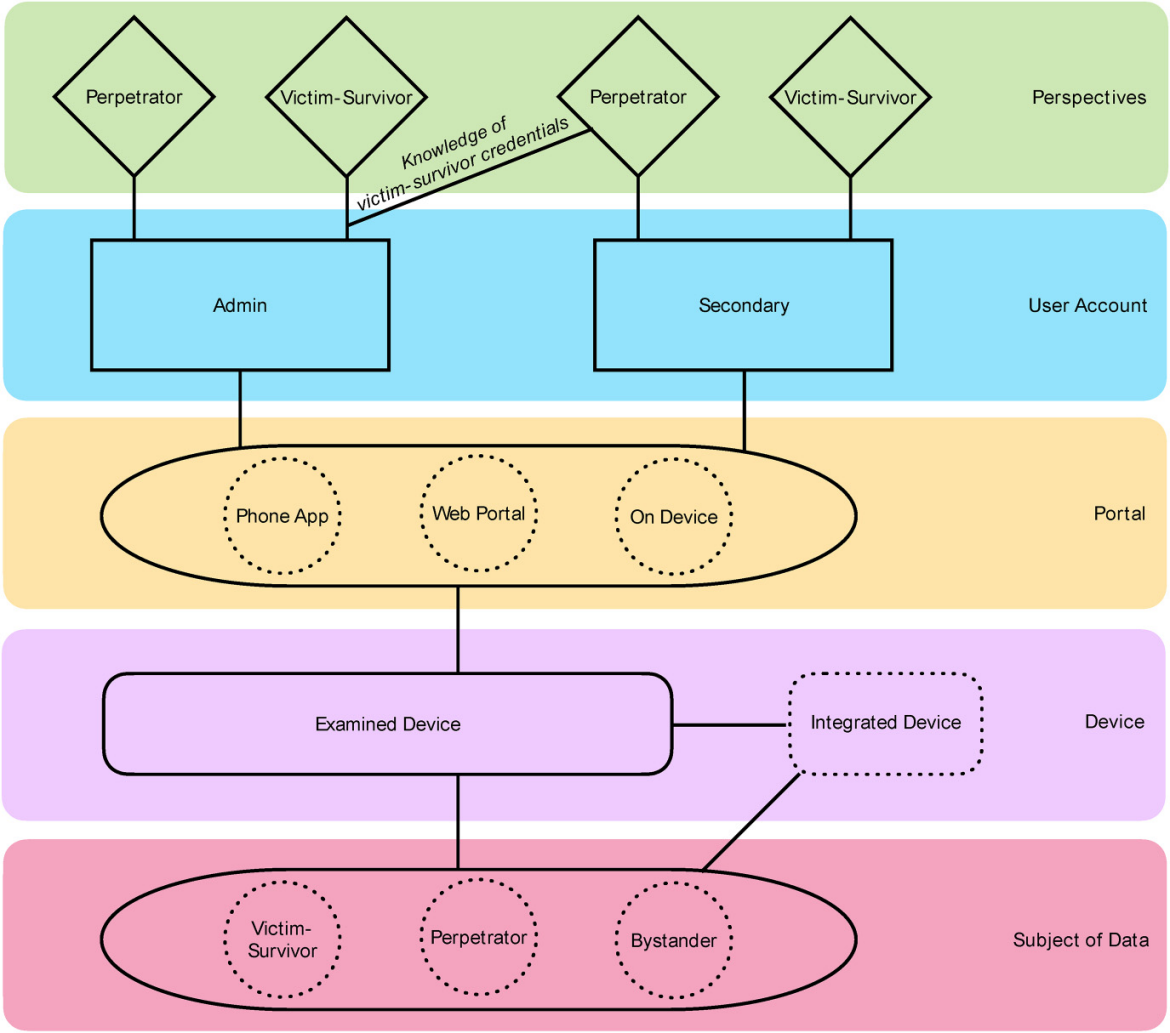
Interactions examined.

The analysis process of many devices involved downloading an associated app onto a smartphone and, for specific items, visiting an online portal where data was being shared (such as live-stream footage from a camera or doorbell). Two smartphones and two laptops were used as access points to the devices. In some cases, these multiple points of access were used to imitate unique users, while in other scenarios, it was assumed that one or more individuals could access devices from numerous points. Multiple accounts in association with each device were set up. This approach allowed the research team to examine how compound users may access the smart home ecosystem through different means (see Figure 1) and was essential to conceiving the variety of ways in which individuals’ interactions with these devices could facilitate or mitigate DFV.

It was assumed that abuse could occur in a variety of circumstances, such as within an “active” relationship, at the point of separation, or beyond separation (Matthews et al., 2017). These distinct phases are significant as they present different avenues, opportunities, and dynamics to exploit smart systems against victim-survivors. It was also considered that the IoT device could be deployed initially by different primary users. Ownership of the IoT device could principally rest with the abuser, or it could be the property of the victim-survivor. Furthermore, we considered that IoT can operate within a “shared” family situation or that the home may have a complicated postseparation arrangement.

Additionally, in juxtaposition to the discrete analysis of individual products, we endorsed interactions between different systems and investigated the effect of device integration. This meant that compatible devices were permitted to sync, allowing us to examine how they shared information. Figure 1 illustrates the examined interactions of victim-survivor or perpetrator perspectives, admin or secondary user account privileges, portals (phone app, web-portal, on-device—where applicable), examined device (and integrated device—where applicable), and subjects of data (victim-survivor, perpetrator, bystander—where applicable). We probed each variation of these possible interactions for every device.

### Ethics and Reporting

Before outlining our findings, we acknowledge several sensitivities around documenting and detailing methods of abuse. It must be considered that sharing evidence between researchers should not inadvertently advertise or expose possibilities for exploitation to DFV perpetrators. Therefore, the following findings will use discretion to balance the need to be comprehensive and specific for research purposes without providing an instruction manual or “how to guide” for abusers.

## Results

This section outlines the findings of our abusability analysis of 13 commercially available IoT systems. Drawing on our enquiry question guide (see Box 1), we tested smart products’ opportunities for (mis)use in the context of DFV. Zooming in on existing safety-by-design principles, such as a device's monitoring capabilities, data collection/sharing practices, and prompts and notification mechanisms, we identified several ways in which design choices embedded within IoT devices create possibilities for abuse. Specifically, we identified seven IoT design features which may facilitate DFV harm. We outlined those risks in turn below. Alongside these observations, we offer redesign suggestions that may foreclose on these abuse possibilities. While the analysis represents a nonexhaustive list of problematic functionalities and design shortcomings, we are confident that the risk vectors we discovered should be factored in by IoT manufacturers and government stakeholders when conceptualizing safety-by-design frameworks for victim-survivors.

Before exploring the aggregate findings further, it is also pertinent to observe that—analogous to Parkin et al. (2019)—our work evidences how “primary” users (i.e., those with admin account credentials) are capable of configuring IoT devices in a way that can exercise power over “secondary” users. This dynamic has clear ramifications for intimate partner violence and is notable for being incessant and producing accompanying flow-on effects throughout the remaining observations. Hence, the designation of user roles and accompanying credential permissions should be accounted for in safety-by-design considerations at a holistic level. Additionally, some vulnerabilities and suggestions for redesign in the context of DFV overlap with elements relevant to general smart home bystanders’ privacy (Thakkar et al., 2022). Consequently, attention should be paid to the opportunity for cocontribution of research across these fields.

### Lack of Visual Reminders of Which Users Have Access to the IoT Device's Functions/Data

#### The Risk

One recurring issue detected across multiple IoT devices, including popular smart home hubs, is that it is often unclear which users have access to the IoT device (either its functions or data). For example, one smart home hub from our sample is designed to be placed within a household and has an in-built camera and microphone. Setting up the device requires users to install an application on their smartphone, and the smart home hub can accept multiple users. Based on default settings, each user can access an all-inclusive list of voice commands made to the hub (i.e., all recordings of all users’ commands) and listen to every recording (“review voice history”). As many users can be added to the account, it is easy to lose track of which users can access the device. This issue is exacerbated as there is no clear and conspicuous indicator of how many profiles have been created. From the perspective of DFV, the critical point is that users of the device can quickly lose sight of what sensitive information other users can access. It is conceivable, for instance, that a victim-survivor may not be aware that a former partner still has remote access to their device and data.

#### Safety-by-Design Suggestion

Providing users with more robust transparency around who has admission to the IoT system's functions and data is likely to support victim-survivors in having greater control over their devices and promote awareness of any legacy permissions granted to abusive or former partners. One potential solution is that manufacturers could present users with a visual representation of who is authenticated to access the device data. This mechanism could be briefly displayed when the device is turned on or when the associated app is opened on a smartphone. The system could remind legitimate users of which other users are on the IoT device network and who has access to the associated data.

### Lack of Visual Reminders of Which Devices Have Access to the Functions/Data

#### The Risk

In the context of DFV, it can often be assumed that abusers may know the usernames and passwords of the victim-survivor. These credentials can therefore be used by abusers to log in to victim-survivors’ smart home technology accounts. Unfortunately, within the sample studied, most of the consumer IoT products did not restrict, log, or note which unique devices were being used to access an account (either an IP, MAC address, or device identifier). This means an abuser could use the credentials of victim-survivors to log in and access smart home technology from an assortment of devices (e.g., personal mobile, work computer) without detection.

#### Safety-by-Design Suggestion

In addition to information about which and how many *users* are connected to the smart device, it should be clear what and how many *devices* are being used to connect. Therefore, it should be clear to legitimate users of IoT devices what other unique devices (such as smartphones, computers, or anything with an “IP” and/or unique “MAC address”) are connecting to their accounts. This process can prevent a perpetrator from using their target's known logins to access IoT devices secretly and without a record of connection. Conspicuous and visible reminders of which devices are being used to access the data and functions of the consumer IoT product would mitigate against both stolen and known credentials being used against someone in a DFV scenario. This security feature is already offered by popular online services such as Gmail or Dropbox and could be encouraged as standard practice within IoT.

### Lack of Visual Indicators of Side-Integration With Other IoT Devices (and What Data and Functions Are Being Shared)

#### The Risk

A common feature of the major smart home hub devices is that they promote “side-integration” with other smart systems (including those developed by “third-parties”). For example, third-party cameras can be “paired” with smart home hubs, allowing these hubs to access the data and functions of the paired devices. “Interconnectivity” is often seen as a desirable feature in the IoT market. However, it can produce unwanted assemblage and information leakage between integrated devices. In one illustration from our sample, a popular fitness tracker “auto-paired” with an iPhone and mirrored the contents of the iPhone's push notifications on the fitness tracker's screen. This enabled the leakage of confidential information from the phone to the fitness tracker (including private SMS contents). Consequently, interconnectivity features may impend the safety of victim-survivors by providing extended opportunities for IoT-enabled TFA. Increasing the threat against victim-survivors, it is often unclear which devices have been coupled with another.

Another example of side-integration within our sample revealed how a smart home assistant could be used to log in to an independent, smart doorbell account. This enabled the live camera footage of the doorbell to be viewed by the home assistant. Once this access was established, the home assistant could also receive alerts when the doorbell device detected motion or had its doorbell pushed. Crucially, there were no conspicuous indicators or reminders within the user interfaces of either the home assistant or the unique doorbell device that any side integration had been established. There were no reminders to the principal smart doorbell user for instance, when the smart home assistant had accessed their live feed or notification that this other device was receiving real-time motion-detection notifications. This type of feature could easily be (mis)used by abusers to continuously collect data (e.g., live camera footage) from a device (e.g., doorbell) without the device owner's (i.e., victim-survivor) knowledge.

#### Safety-by-Design Suggestion

Side-integration is often actively encouraged by IoT devices, and, therefore, likely to be actionable through “set and forget” configuration processes. This means it is often unclear on the user interfaces that the devices are side-integrated, thus permitting the inconspicuous sharing of data and functions with unknown or unseen users (on an unknown number of occasions). To help overcome the potential for devices to integrate in a manner which facilitates IoT-enabled abuse, it is recommended that “set and forget” interconnectivity is replaced with ongoing notification and/or reauthorization protocols. For example, IoT manufacturers could mitigate against surreptitious (mis)use of integration features by producing more visual reminders on user interfaces that prompt owners to recognize and reflect on device interconnectivity. The notifications displayed on Apple products in the vicinity of AirTags are an example of such reminders. Easy-to-read data visualizations which show a complete overview of device network integration could be displayed on screen-enabled products, (e.g., presenting directly on device screens and/or when opening associated apps/portals). These visualizations would act as a reminder to users about which devices are integrated and what that means in terms of where the data is being shared and with whom. Consequently, network overview visualizations could also help address the above issues surrounding the lack of notification about which *users* and *devices* have network access.

### Lack of Noise or Light Indicators to Indicate the Immediate Physical Environment When the Sensors Are Active

#### The Risk

Not all IoT devices feature noise or light indicators that their sensors are live and capturing information. For example, a smart doorbell in our sample allowed users to view their livestream camera footage without the device LEDs being on. The doorbell, thus, does not clearly signal to those in the camera's environment that the device is active. Considering the increasing ubiquitousness of IoT products, it is easy to anticipate how such a lack of warning can be (mis)used to surreptitiously watch others, particularly when the sensors may be activated from a remote location. This is a privacy concern for all persons exposed to these potential intrusions, but specifically, in the context of DFV, the capacity for surveillance afforded by devices without active indicators can significantly extend abusive behavior (e.g., monitoring/stalking).

#### Safety-by-Design Suggestion

A safety-by-design principle that IoT devices could apply is that those near or around the physical devices should have clear and conspicuous indicators that the device is collecting information. The use of LED lights or noise-emitting notifications to indicate that sensors are active—and therefore listening, streaming, recording, or otherwise capturing information—could mitigate the probability of IoT devices being deployed for covert surveillance. With this approach, it is important to recognize a broad definition for when devices are “collecting” information and sensors are “active.” This will reduce instances such as livestream footage being accessible without indication since livestream footage is not classified as being “collected” under a narrow definition that relates to the footage being “recorded” and/or “stored” for later consumption. Similarly, devices which include “always on” sensors as part of their basic functioning should have indicators for both passive and dynamic sensor activation. Smart home security cameras could have a correlating “always on” LED light to indicate passive camera functioning and a different indicator (e.g., noise emission) for dynamic interactions such as camera feed being accessed. Clear, and permanent indications of sensor functions should also be included on the physical device. For example, pictograms signifying the types of sensor function/s could be cast or molded into exterior casings of devices.

### Lack of Accessible Log Files of Which Users and Devices Have Accessed the IoT Functions/Data

#### The Risk

Certain cameras and smart home hubs (with embedded cameras) display visual indicators that someone is currently “watching live.” A particular hub in our sample, for instance, has a blinking green LED on the device indicating that someone is presently viewing the livestream camera footage, and will also have an on-screen notification saying that the camera is active. However, once the “watching live” function ceases, all notifications of camera activity disappear. Additionally, no record that the viewing occurred is left. Individuals with this smart home hub in their household thus have no immediate, on-device means of checking if their camera was accessed, by *whom* or *when*. This is concerning for those in coercive and abusive relationships as it allows opportunities for controlling abusers to conduct surveillance of a home without leaving clear, conspicuous traces.

#### Safety-by-Design Suggestion

The safety-by-design principle of providing “log files” of *which users* and *which devices* have accessed the IoT functions or data could address many of the issues surrounding surreptitious (mis)use. Greater transparency could be added to IoT devices about *who*, *what*, and *when* various users and devices accessed data or functions, preventing unrecorded instances of (mis)use, such as using cameras to control and surveil victim-survivors. This step could limit the ability of the devices to be used for stealthy surveillance.

Manufacturers should also anticipate that IoT can be placed in an individual's home or personal space for coercion or unwanted surveillance. Providing accessible log files to all users could offer a mechanism of transparency and confidence that devices are not being used against individuals without their knowledge. Additionally, users could cross-check who or what has been accessing their data (or device's functions), and thus quickly identify unauthorized, unwanted, or illegitimate users.

Moreover, the implementation of accessible log files could be a valuable asset for demonstrating a “course of conduct” around nonconsensual surveillance or “coercive control” within the context of legal proceedings. Most countries have offences against “stalking” that are relevant in the context of DFV (McMahon et al., 2019), and several jurisdictions—such as England and Wales or Tasmania (Australia)—have criminalized the offence of coercive control (McGorrery & McMahon, 2019). In a jurisdiction such as Victoria (Australia), stalking is prosecuted after demonstrating a “course of conduct” of “tracing the victim's … use of the internet or of email or other electronic communication” (Crimes Act 1958 (Vic) s21A). Making IoT device log files available to victim-survivors could offer digital evidence to demonstrate harmful courses of conduct undertaken by abusers and make excessive and unwanted monitoring visible to legal proceedings. In this respect, IoT manufacturers can introduce changes that could support victim-survivors in the criminal justice system.

### Weak Protections on Associated Online Portals

#### The Risk

In addition to users accessing data or functions via a smartphone app, many prominent IoT devices also allow users to review and control functionalities via separate web-accessible portals. These online portals are an additional point of vulnerability which can be (mis)used to remotely access device data and functions. None of the devices within our sample that provided URL-accessible portals had accessible file logs for the online portals, and none provided notifications to legitimate users that the online portal was being used. This means abusers could monitor and interfere with victim-survivor IoT device activities both stealthily and without physical proximity.

Due to their low prominence, it is also likely that many IoT device users are unaware that their usage data is viewable via online portals. This provides additional opportunities for an abusive partner to monitor their target without the legitimate users’ knowledge. This method of surveillance can also be deployed without the use of IoT product-specific applications, the presence of which could act as potential evidence against the perpetrator.

#### Safety-by-Design Suggestion

Manufacturers should aim to promote transparency and prevent remote (mis)use of IoT systems, by ensuring any login attempts through online portals cause alerts informing the relevant account holder/s that this event has taken place. Furthermore, the login process for accessing online portals could require TFA or multifactor authentication, or at least notify the users of the IoT device that this data is being looked at via an online portal. This type of step could either prevent surreptitious monitoring of victim-survivors by a current or former partner, or, at the very least, create a record of behavior for use in criminal proceedings.

### Many Devices Are Vulnerable to “Password-Reset Attacks”

#### The Risk

Many of the IoT devices examined were vulnerable to a “password-reset attack,” whereby an illegitimate user can unilaterally seize control over the data and functions of an IoT device by impersonating users and requesting password changes. Password-reset attacks involve using the legitimate password recovery system to hijack an account registered to an IoT device. This process often consists of the attacker knowing their targets’ personal email login credentials, which, unfortunately, is very likely within the context of DFV. Password-reset attacks may act as a mechanism for abusers to further extend their own control and capacity to enact TFA, as well as diminish victim-survivors’ agency.

#### Safety-by-Design Suggestion

Manufacturers of IoT should consider that email logins are frequently compromised in the context of DFV, and abusers could use this to seize control over smart products used by victim-survivors. In such scenarios, single-factor authentication and relying solely on email to reset the password may be insufficient. Instead, the reset process should involve deploying multifactor authentication or similar steps, such as verifying phone calls to ensure only the legitimate user can reconfigure the password. Additionally, options to select multiple points of notification about password resets being requested (e.g., phone and email notifications) would make it more difficult for abusers to obscure these attacks (i.e., by deleting password reset emails while digitally trespassing upon victim-survivor's email accounts). Such techniques could prevent abusive partners from remotely taking control of IoT devices used by victim-survivors.

Overall, the findings presented here are mere examples of anticipating (mis)use in the context of DFV. Our study does not claim to offer an exhaustive list of abuse possibilities or safety-by-design considerations, and there are many other factors to consider in addressing DFV. Nonetheless, the findings highlight that IoT manufacturers need to begin anticipating how abusive perpetrators of DFV may utilize their devices, and the above observations reflect the kind of threat scenarios they must consider. The following section will unpack the broader implications and possibilities for redesigning IoT to encourage safety and confidence for victim-survivors of DFV. We also address the long-term considerations necessary for ensuring the ongoing, healthy digital participation of victim-survivors of DFV.

## Discussion

In this study, we used a heuristic walkthrough method to conduct an abusability analysis of 13 commercially available IoT systems to probe opportunities for (mis)use in the context of DFV. The insight derived from our study is two-fold. Firstly, we observed that IoT designs generate permission hierarchies that offer avenues for abusive behavior, supporting principal work by Parkin et al. (2019). Additionally, we identified seven IoT design features that may facilitate DFV harms and must be addressed in future “safety-by-design” approaches by industry or government stakeholders. These include:

1. Lack of visual reminders of which users have access to the IoT device's functions/data
2. Lack of visual reminders of which devices have access to the functions/data
3. Lack of visual indicators of “side-integration” with other IoT devices (and what data and functions are being shared)
4. Lack of noise or light indicators to indicate the immediate physical environment when the sensors are active
5. Lack of accessible logs of which users and devices have accessed the IoT functions/data
6. Weak protections on associated online portals
7. Many devices are vulnerable to “password-reset attacks.”

Secondly, we propose amendments for each uniquely identified abusable feature to return or increase victim-survivor control over IoT systems. These suggestions notably center around enhanced transparency regarding IoT device functionality. In particular, there is an emphasis on ensuring that users are aware of who can access the IoT device functions and data and can identify when and where device access has occurred. The suggested redesigns essentially offer a safety net for those users who may be denied autonomy over their technology engagement, especially concerning preserving the integrity of password/login information. In sum, our findings demonstrate ample opportunities for safety-focused IoT (re)design to assist in curbing exploitative use and offering increased protection for victim-survivors of DFV.

However, a few further challenges present themselves that would need to be navigated and countenanced before expecting widespread industrial change to IoT design. The first is cost. Factoring in the implications for victim-survivors will add additional expenses to any development process. Plus, attempting to retrofit software or hardware may also carry financial outlays. Some amendments are cheap and straightforward to implement (i.e., would involve only minor additional coding tasks and could be included in future system updates), so the problem of cost should not be overstated. But it will be hard to convince many manufacturers to undertake a consideration that adds overhead to their production process.

To counteract the above, manufacturers could be encouraged to realize the social benefits of this pursuit. Women comprise a large portion of victim-survivors of DFV and are a vital customer base. For women to perceive smart systems as safe and secure will have enduring market relevance. The multinational technology corporation IBM (2020) has already produced five design principles for combatting domestic and family abuse, while the cybersecurity and antivirus provider Kaspersky supports the antistalking efforts of the European Network for the Work with Perpetrators of Domestic Violence. IBM further leads an Institute of Electrical and Electronics Engineers Working Group to address technology-facilitated interpersonal control (P2987). These efforts highlight that some companies see the value and need for these safety-by-design efforts.

Other challenges emerge when considering that the consumer IoT industry comprises companies of various sizes, capacities, and countries of origin. The IoT sector is expanding, with new vendors and manufacturers emerging rapidly. This “cottage” industry of new operators sits alongside the IoT products produced by major tech giants such as Apple, Google, and Amazon, which often dominate the market.

Significant DFV organizations, such as the National Network to End Domestic Violence (NNEDV) in the United States, have already established connections and partnerships with larger tech corporations, including Google and Apple (NNEDV, 2020). Therefore, some major players could be convinced and inclined to make the necessary adjustments. In the wake of the controversy surrounding the stalking potential from “AirTags,” Apple publicly insisted that they are open to discernment from DFV organizations (Fowler, 2021) and most recently teamed up with Google to define industry specifications to address unwanted tracking (Apple, 2023). If such collaborations are implemented in meaningful ways, subsequent design changes could have an extensive reach in a relatively short period of time.

Ensuring that small or emerging manufacturers participate in redesigns is a more significant challenge. It is harder to identify and reach out to all the emerging consumer IoT manufacturers, and they may be less receptive to the need to take on extra production burdens. Should manufacturers choose not to adjust and self-regulate, the IoT sector may be mandated to make these changes using policy tools such as legislation. Although, as indicated below, this approach has complexities.

While it is hypothetically conceivable that government authorities compel manufacturers to abide by certain product safety standards—much like there are current mandatory standards enforced by bodies such as the Australian Competition and Consumer Commission (ACCC, 2020) for items like baby walkers, cosmetics, swimming pools, and cigarettes—the introduction of voluntary codes of practice for IoT manufacturers in the United Kingdom and Australia suggests a general reluctance to compel engineering changes legally (Department for Digital, Culture, Media and Sport 2018; Department of Home Affairs, 2020).

In these contexts, where “hard” government regulation is unlikely, industry bodies such as the IoT Alliance Australia could be a helpful intermediary and pressure point for encouraging appropriate industry standards. Simultaneously, the ongoing creation and dissemination of recommended and best-practice principles (see, e.g., Hussain et al., 2022; Institute of Electrical and Electronics Engineers (IEEE) Standards Association, 2021) may continue to increase awareness and foster voluntary involvement among harder-to-reach audiences.

Additional compliance problems emerge when it is considered that IoT manufacturers are international organizations. It may be challenging to leverage companies that operate across borders to comply with safety-by-design demands that may only arise in particular markets. However, global IoT advocacy organizations such as the IoT World Alliance or the IoT Consortium could play a role in encouraging international norms (Datta Burton et al., 2021). Likewise, crucial international trade actors such as the European Union could lead in requesting higher standards of safety.

Another international dimension of IoT manufacturers that needs to be considered is that IoT manufacturing can be a global process. For example, the primary software may be developed in one nation, while major components and hardware could be developed elsewhere. Likewise, certain companies may purchase devices from one jurisdiction and then rebadge them or sell them via an entirely different company. These complications in the production chain can create a further challenge for regulating IoT and advancing safety-by-design frameworks.

Ultimately, there are many hurdles to safeguarding IoT for victim-survivors of DFV. As outlined above, IoT in the context of DFV has several unique safety risks, and there are many ways that design features can be subverted for abuse. IoT manufacturers, government authorities, and industry bodies, therefore, ought to be part of public strategies to combat DFV-related TFA. The whole sector must begin to rely on abusability analysis mechanisms, such as those outlined here, to anticipate exploitation and to initiate safety-by-design standards that can foreclose any identified risks. Only a proactive, forward-thinking IoT market will prevent DFV perpetrators from (mis)using their products; by making better, less harmful design choices that can inadvertently impact vulnerable groups and communities and make them safer and more secure moving forward.

### Limitations

We are aware that our research may have several limitations. First, this research only reflects a narrow snapshot of the practices that are being deployed in the IoT market. Second, system updates mean that IoT devices already “in the field” can change their functionality. Hence, any specific claims about devices reflect only how they performed in early 2020, and technical observations may have been addressed, changed, or otherwise modified by the manufacturer.

Third, perpetrators in the context of DFV may envision new or different ways to leverage consumer IoT for abusive ends. Accordingly, considerations of abusability and safety by design should be perceived as an iterative process. Attention must be paid to the idea that safety-by-design changes, such as those proposed here, may also be exploited by perpetrators.

Fourth, it is conceivable that some design changes may inadvertently be to the detriment of victim-survivors (Matthews et al., 2017). For instance, some victim-survivors may want to use IoT as a covert surveillance aid for evidence gathering against a perpetrator. Some of the above-recommended changes may undermine this capacity. On balance, more transparency is likely to favor victim-survivors in many situations. Still, this may not always be the case and vendors must be alert to the possibility that perpetrators can also misuse transparency features. For this reason, ongoing changes to IoT design would need to be monitored and made in consultation with DFV experts or frontline support workers who could provide strong evidence and feedback relating to the lived experiences of victim-survivors and what best suits their needs.

Lastly, IoT redesign is only a partial aide to combatting DFV-related TFA and should always be part of a broader public safety strategy that incorporates more direct support for victim-survivors along with wider gender equality-focused social reforms in society.

## Conclusion

This article put forward an abusability analysis which revealed seven design features in a sample of 13 consumer IoT devices that inadvertently facilitate their exploitative potential in the context of DFV. To date, IoT manufacturers and government authorities have not adequately conceptualized DFV as an IoT threat. However, as consumer IoT devices continue to proliferate in market penetration and the variety of devices placed in homes, this only increases the likelihood and danger that IoT will be used abusively. Therefore, it is imperative that government authorities, industry bodies, and IoT practitioners urgently conceptualize DFV as a key IoT issue and factor it into safety-by-design frameworks, codes of practices, and other forms of security guidelines and threat models. Where possible, regulation should consider enforcing IoT manufacturers to consider the needs of victim-survivors of DFV. However, it is also critical to recognize that victim-survivors’ conditions may change or vary over time, making ongoing consultation between DFV experts and the IoT industry necessary. Safety-by-design for victim-survivors of DFV is just one part of a broader effort to begin safeguarding the IoT for a vulnerable and sizable population.
